# Mortality After Hip and Spine Fractures in Patients With End-Stage Kidney Disease: A Systematic Review and Meta-Analysis

**DOI:** 10.7759/cureus.49636

**Published:** 2023-11-29

**Authors:** Yoshinosuke Shimamura, Yasutaka Kuniyoshi, Hiroshi Ueta, Takamasa Miyauchi, Mari Yamamoto, Yasushi Tsujimoto

**Affiliations:** 1 Nephrology, Teine Keijinkai Medical Center, Sapporo, JPN; 2 Pediatrics, Tsugaru Hoken Kensei Hospital, Hirosaki, JPN; 3 Anesthesiology and Critical Care, Kobe City Hospital Organization, Kobe City Medical Center General Hospital, Kobe, JPN; 4 Nephrology and Hypertension, St. Marianna University School of Medicine, Kawasaki, JPN; 5 Nephrology, Chubu Rosai Hospital, Nagoya, JPN; 6 Medicine, Oku Medical Clinic, Osaka, JPN; 7 Systematic Reviewers, Scientific Research WorkS Peer Support Group (SRWS-PSG), Osaka, JPN

**Keywords:** spine fracture, hip fracture, mortality, kidney replacement therapy, end-stage kidney disease

## Abstract

Fractures represent a major cause of disability in the elderly, and patients with fractures exhibit a higher mortality rate than those without. Fractures are also an important health problem among patients with end-stage kidney disease (ESKD) requiring hemodialysis, peritoneal dialysis, or kidney transplantation. To the best of our knowledge, no study in the literature has yet quantitatively summarized the mortality rates, and a summary of evidence on post-hip and spine fracture mortality in patients with ESKD is lacking.

The purpose of this study is to quantitatively evaluate the mortality rate, one-year mortality rate, and five-year mortality rate after hip and spine fractures in patients with ESKD receiving kidney replacement therapy.

The MEDLINE, Cochrane Central Register of Controlled Trials (CENTRAL), Embase, and ClinicalTrials.gov databases were comprehensively searched for reports on mortality rate and time-period mortality in patients with ESKD after hip or spine fractures up to June 2022. Prospective and retrospective cohort studies, as well as case series involving four or more patients, were included. Pooled mortality rate, one-year rate, and five-year mortality rate with 95% confidence intervals (CIs) were examined using a random-effects model. The risk of bias was assessed using the Joanna Briggs Institute (JBI) Prevalence Critical Appraisal Tool. Additionally, heterogeneity between studies was evaluated.

A total of 26 studies were included in this meta-analysis. The one-year and five-year mortality rates after hip and spine fractures were 215.35-774.0 per 1,000 person-year and 148-194.1 per 1,000 person-year, respectively. After hip fractures, the one-year mortality rate was 27% (95% CI: 18-38%, I^2^ = 98%), whereas the five-year mortality rate was 56% (95% CI: 41-71%, I^2^ = 99%). After spine fractures, the one-year mortality rate was 10% (95% CI: 4-17%, I^2^ = 70%), whereas the five-year mortality rate was 48.3%.

The post-fracture mortality rate was high in patients with ESKD, particularly within one year after the occurrence of fractures. Additionally, the five-year mortality rate after hip femoral or spine fractures was high at approximately 50%.

## Introduction and background

Fractures represent a major cause of disability in the elderly, and the risk of fractures, including inadvertent falls, frailty, osteoporosis, and menopause, increases with age [1]. More than two million osteoporosis-related fractures have been estimated to occur in the United States, with spine and hip fractures accounting for 27% and 14%, respectively [2]. Patients with fractures exhibit a higher mortality rate than those without [3-5]. In particular, the one-year mortality rate after hip fractures has been reported to be approximately 3.7 and 2.8 times higher in men and women with fractures, respectively, than in non-fracture patients [5].

Fractures are also an important health problem among patients with end-stage kidney disease (ESKD) requiring hemodialysis, peritoneal dialysis, or kidney transplantation [6,7]. Compared to the general population, patients with ESKD have a two- to four-fold increased risk of hip and spine fractures [8-11]. Such an excessive fracture risk in patients with ESKD is likely attributable to underlying mineral metabolism abnormalities that lead to renal osteodystrophy, as well as an increased fall risk from neuromuscular impairments [12]. In particular, hip fracture has severe consequences in patients with ESKD, including an increased risk of hospitalization, reduced quality of life, loss of independence, and death [12-17].

Previous cohort studies have reported one-year mortality rates ranging from 14% to 43% after hip fractures in patients with ESKD [15-17]; however, the reported rates widely vary across studies, possibly owing to differences in sample sizes, patient demographics, or kidney replacement therapy modalities. To the best of our knowledge, no study in the literature has yet quantitatively summarized the mortality rates, and a summary of evidence on post-spine fracture mortality in patients with ESKD is lacking. Therefore, we conducted a systematic review and meta-analysis to provide precise estimates of mortality after hip and spine fractures in patients with ESKD who are undergoing kidney replacement therapy.

## Review

Methods

Compliance With Reporting Guidelines

This systematic review was conducted in accordance with the Preferred Reporting Items for Systematic Reviews and Meta-Analyses (PRISMA) statement [18]. We confirmed that our systematic review was PRISMA-compliant by consulting the 2020 PRISMA checklist (Appendix Table 3). The prespecified protocol can be accessed at https://www.protocols.io/view/systematic-review-and-meta-analysis-of-incidence-a-capzsdp6. While this study involved human participants, ethical approval from the institutional review board was not obtained, given that ethical approval was sought by the individual original studies included in the systematic review. Informed consent was obtained from all individual participants included in each study prior to their study participation. All authors received no specific grant from any funding agency in the public, commercial, or not-for-profit sectors for this research.

Eligibility Criteria for the Included Studies

The eligibility criteria were as follows:

(ⅰ) Study design: prospective and retrospective cohort studies and case series involving four cases or more (a case referred to a patient with ESKD after hip or spine fracture)

(ⅱ) Study population: patients with ESKD, defined as the requirement for hemodialysis, peritoneal dialysis, or kidney transplantation, irrespective of primary disease

(ⅲ) Outcome: studies reporting on mortality or mortality rate

(ⅳ) Time: outcome reported at least one month after hip or spine fracture

Studies were eligible irrespective of publication status, follow-up period, language, age, sex, race, or surgery status. However, studies that did not recruit or were withdrawn from ClinicalTrials.gov, case reports describing three or fewer cases, animal and laboratory studies, and literature reviews were excluded.

Outcome Measures

The primary outcomes evaluated were the (i) mortality rates after hip and spine fractures, (ii) one-year mortality rate after hip and spine fractures, and (iii) five-year mortality rates after hip and spine fractures. The diagnoses of hip and spine fractures set by the original authors included ascertainment from the presence of a corresponding International Classification of Diseases, 9th Revision, code in a hospital billing claim.

Search Methods for Study Identification

Electronic searches: The Cochrane Central Register of Controlled Trials (CENTRAL), MEDLINE (via Ovid), and Embase (via ProQuest) databases were searched for relevant studies on June 23, 2022. The search results were filtered for the prognostic factors reported by Wilczynski et al. [19] (Appendix Tables 4-8).

Searches of other resources: The World Health Organization’s International Clinical Trials Registry Platform (ICTRP) Search Portal and ClinicalTrials.gov registry were also searched to identify completed unpublished studies and to investigate reporting bias. Furthermore, the references of extracted studies and international guidelines were checked, and the authors were contacted if the extracted studies lacked the necessary data. The detailed search strategies are described in Appendix Tables 7, 8.

Data Collection and Analysis

Study selection: Two out of the four reviewers, Yoshinosuke Shimamura (Y.S.), Hiroshi Ueta (H.U.), Takamasa Miyauchi (T.M.), and Mari Yamamoto (M.Y.), independently screened the titles and abstracts identified during the search. A predefined protocol was followed in screening the abstracts and full texts, and predefined criteria were used in the registered protocol. All extracts from the reviewers were subjected to a full-text review; subsequently, they independently determined whether the full text should be included in the review. The first author (Y.S.) checked all included studies and applied the exclusion criteria for all records subjected to the full-text screening procedure; hence, the decision did not differ systematically. The original authors were contacted if the study had an abstract only or if it was unclear whether the study met the review criteria. Any disagreement was resolved through discussion between the two reviewers; if an agreement could not be reached, a third reviewer, Yasutaka Kuniyoshi (Y.K.) or Yasushi Tsujimoto (Y.T.), acted as an arbiter.

Data extraction and management: Two reviewers independently performed the data extraction, and any disagreement between the two reviewers was resolved through discussion. A third reviewer was involved in the discussion, where necessary, and the original authors were contacted. The one-year and five-year mortality rates were extracted; conversely, mortality rates at less than one year were not extracted. Additionally, data were extracted when the studies reported mortality rates at more than one year and less than five years (e.g., two-year mortality rate), even if they did not report either a one-year or five-year mortality rate. In such cases, data on the outcome at less than three years were considered as the one-year mortality rate, whereas data on the outcome at three to five years were considered as the five-year mortality rate. A pre-checked data extraction form with 10 randomly selected studies was utilized. The mortality rate was calculated as described in respective studies.

Assessment of the risk of bias of included studies: Two reviewers independently assessed the risk of bias in each study using the JBI Prevalence Critical Appraisal Tool [20,21]. The following domains were assessed:

1. Was the sample frame appropriate to address the target population?

2. Were the study participants sampled appropriately?

3. Was the sample size adequate?

4. Were the study participants and setting described in detail?

5. Was the data analysis conducted with sufficient coverage of the identified sample?

6. Were valid methods used for the identification of the condition?

7. Was the condition measured in a standard and reliable manner for all participants?

8. Was there appropriate statistical analysis?

9. Was the response rate adequate? If not, was a low response rate managed appropriately?

Any disagreement was resolved through discussion among the reviewers; if an agreement could not be reached, a third reviewer acted as an arbiter. In this study, the overall risk of bias was calculated as the number of “yes” responses for each domain divided by the total number of domains and was expressed as a percentage. The overall risk of bias was interpreted according to the calculated percentage as follows: <50%, high risk of bias; 50-80%, moderate risk of bias; >80%, low risk of bias [21].

Measures of the Treatment Effect

In this study, both the incidence rate (measured as the number of incident cases per measure of exposure) and incidence proportion (measured as the number of incident cases over a specified period) were determined with 95% confidence intervals (CIs) and 95% prediction intervals. The between-study variance was estimated using tau2 statistics, which supply a logit scale measure of between-study variance, represented in a more readily interpretable way by 95% prediction intervals.

Data Synthesis

A single-arm analysis was conducted. Percentages, means, and standard deviations were calculated for categorical variables. The pooled mortality rate and mortality were calculated for patients with ESKD after hip fractures and those with ESKD after spine fractures. A random-effects model (DerSimonian and Laird approach) was used for pooled estimates to consider the variance between and among the studies. Statistical analyses were performed using R software (R Development Core Team 2019), with meta version 4.15-0 and metaphor version 2.4-0.

*Dealing With Missing Value*s

For dropouts, imputation was not performed in accordance with the recommendations by The Cochrane Handbook [22]. A meta-analysis was conducted on data presented by the original authors, and any missing values or summary statistics were not complemented.

Heterogeneity Assessment

Heterogeneity was examined via visual inspection of the forest plot and calculation of I^2^ statistics (I^2^ values of 0-40%: “might not be important”; 30-60%: “may have moderate heterogeneity”; 50-90%: “may have substantial heterogeneity”; 75-100%: “considerable heterogeneity”). If heterogeneity was detected (I^2^ > 50%), its plausible cause was verified. The I^2^ statistics were calculated using the Cochrane chi-squared test (Q-test), and statistical significance was set at P < 0.10.

Subgroup Analysis

Considering that the present study aimed to identify the plausible causes of heterogeneity, the following prespecified subgroup analyses of primary outcomes were planned: sex (men vs. women), race (Black vs. non-Black), presence or absence of cardiovascular diseases (hypertension, coronary artery disease, congestive heart failure, cerebrovascular disease, and peripheral vascular disease), presence or absence of diabetes mellitus, type of kidney replacement therapy (hemodialysis, peritoneal dialysis, and kidney transplantation), and participants’ age category (≥75 years vs. <75 years).

Sensitivity Analysis

To confirm the robustness of the main results, a prespecified sensitivity analysis was conducted on the primary outcomes, excluding the outcome data other than the one-year and five-year mortality rates.

Assessment of Reporting Bias

The ICTRP and ClinicalTrials.gov were searched for studies that were completed but have not yet been published. Potential publication bias was assessed via visual inspection of funnel plots and Egger’s test.

Results

Literature Search

**Figure 1:**
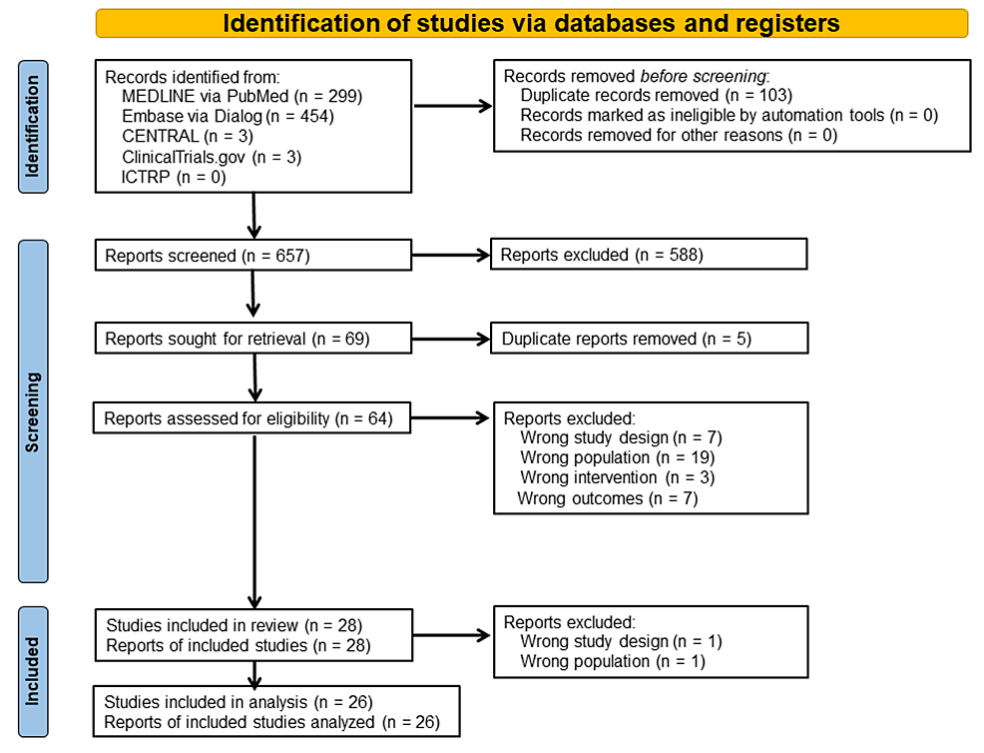
Flowchart of the selection of studies included in the meta-analysis

After duplicate removal, a total of 657 records were identified through a systematic search in MEDLINE, Embase, CENTRAL, ClinicalTrials.gov, and ICTRP. Among these, 69 reports were retrieved for full-text review; however, five reports were excluded because of duplicate publications, resulting in 64 eligible reports based on the inclusion criteria. Furthermore, seven studies with incorrect study designs, 19 studies with incorrect study populations, three studies with incorrect interventions, and seven studies with incorrect outcomes were excluded. Notably, the study by Yuan et al. [23] was excluded from the main analysis because it was a letter article, and the single-center cohort study by Iseri et al. [24] was also excluded because it primarily involved patients with malnutrition, inflammation, and atherosclerosis syndrome and did not provide details on the status of hip and spine fractures in participants. Finally, 26 studies were included in the meta-analysis (Figure 1).

Study Characteristics

Table 1 summarizes the clinical characteristics of the included studies. Overall, the studies were published from 1994 to 2022. Eleven studies were conducted in European countries [25-35], whereas the other studies were from the USA [14,36-39], Japan [40-42], Taiwan [16,17], Canada [43], South Korea [44], and Turkey [45]. Additionally, 17 studies [13,14,16,17,25-27,29-31,33,35-37,40,44,46] were conducted in multicenter settings, and the number of participants considerably varied from 12 to 610,524. The longest follow-up duration was five years, and the participants’ mean age ranged from 39 to 82.3 years. Out of the 26 studies, 20 investigated the prognosis of patients with hip fractures [14,16,17,27-29,31,32,34-41,43-46], and two [30,42] examined the prognosis of patients with spine fractures, whereas four [25-27,37] assessed the prognosis of both hip and spine fractures. Nine studies reported the surgery status for hip or spine fracture [17,28,31,32,37,38,40,43,44]. For hip fracture, five studies [17,31,38,44,45] included both patients with intracapsular and those with extracapsular hip fractures, and three studies [28,41,43] only included patients with intracapsular hip fractures. However, six other studies did not report the type of hip fracture [25,29,37,39,40,46].

**Table 1:** Summary of participant characteristics ^a^Standard deviation ^b^Interquartile range ^c^Range AU, Australia; CS, cohort study; CVD, cardiovascular diseases; DM, diabetes mellitus, EU, Europe; HD, hemodialysis; MC, multi centers; N/A, not available; NZ, New Zealand; PCT, prospective cohort study; PD, peritoneal dialysis; PY, person-year; RCT, retrospective cohort study; SC, single center

First author and publication year	Study design	Country	Follow-up (years)	Setting	Sample size (n)	Age	Male (%)	Modality of renal replacement therapy (HD: PD: transplant) (%)	DM (%)	CVD (%)	Surgery for hip or spine fracture (%)	Prior fracture (%)
Tentori 2014 [13]	RCS	EU, AU, NZ, Japan, and USA	1.6	MC	36,337	EU, AU, and NZ: 74 (65 to 79)^b^; Japan: 68 (60 to 76)^b^; USA: 71 (59 to 80)^b^	45	100:00:00	34	N/A	N/A	3
Mittalhenkle 2004 [14]	RCS	USA	N/A	MC	7,636	71.93 (11.36)^a^	41	100:00:00	50	43	N/A	N/A
Lin ZZ 2014 [16]	RCS	Taiwan	4.1	MC	51,473	N/A	39	97:03:00	60	43	N/A	4
Lin JCF 2015 [17]	RCS	Taiwan	N/A	MC	2,680	74.88 (7.05)^a^	37	100:00:00	43	38	100	N/A
Ferro 2015 [25]	CS	UK	4.7	MC	836	55.27 (11.92)^a^	55	0:01:40	25	7	N/A	5
Iseri 2020 [26]	RCS	Sweden	4.8	MC	3,992	53 (42 to 62)^b^	65	0:01:40	18	N/A	N/A	6
Iseri 2021 [27]	RCS	Sweden	N/A	MC	642	76 (68 to 81)^b^	59	100:00:00	N/A	N/A	N/A	N/A
Kalra 2006 [28]	RCS	UK	N/A	SC	18	71 (52 to 83)^b^	33	100:00:00	N/A	N/A	39	N/A
Arnold 2015 [29]	RCS	UK	N/A	MC	836	N/A	N/A	0:01:40	N/A	N/A	N/A	N/A
Goto 2019 [30]	PCT	Netherlands	0.5	MC	196	75.3 (6.9)^a^	53	75:25:00	11	43	N/A	N/A
Orabona 2019 [31]	PCT	Italy	1	MC	64	76.2 (53 to 94)^b^	31	100:00:00	N/A	N/A	100	N/A
Apostolopoulos 2021 [32]	RCS	Greece	0.3	SC	20	N/A	N/A	100:00:00	N/A	N/A	100	N/A
Iseri 2020 [33]	RCS	Sweden	2.2	MC	9,714	68 (56 to 76)^b^	67	66:34:00	27	N/A	N/A	8
Wu 2021 [34]	PCT	UK	1	SC	397	83.5 (9.2)^a^	47	100:00:00	N/A	N/A	N/A	N/A
Iseri 2020 [35]	RCS	Sweden	2.2	MC	9,714	68 (56 to 76)^b^	67	66:34:00	27	N/A	N/A	8
Kaneko 2007 [36]	CS	USA	3.3	MC	7,159	N/A	52	100:00:00	33	60	N/A	N/A
Beaubrun 2013 [37]	RCS	USA	N/A	MC	610,524	N/A	N/A	N/A	N/A	N/A	N/A	N/A
Tierney 1994 [38]	RCS	USA	4	SC	12	55 (26 to 86)^b^	67	58:17:25	67	17	100	8
Toomey 1998 [39]	RCS	USA	5	SC	15	39	80	100:00:00	N/A	N/A	100	N/A
Wakasugi 2020 [40]	RCS	Japan	5	MC	237,064	73.3 (11.1)^a^	43	100:00:00	39	35	N/A	0
Sakabe 2006 [41]	RCS	Japan	5	SC	62	67.7 (41 to 90)^b^	34	100:00:00	31	N/A	94	N/A
Maeno 2009 [42]	PCT	Japan	4.5	SC	635	68.8 (10.2)^a^	58	100:00:00	29	N/A	N/A	100
Ouellet 2008 [43]	RCS	Canada	10.6	SC	60	N/A	N/A	N/A	N/A	N/A	N/A	N/A
Jang 2020 [44]	RCS	Korea	3	MC	19,915	N/A	43	95:05:00	N/A	N/A	100	N/A
Karaeminogullari 2007 [45]	RCS	Turkey	1	SC	40	57 (19 to 81)^c^	N/A	N/A	25	N/A	73	0
Arnold 2018 [46]	CS	UK and USA	1	MC	30,095	England: 48 (38 to 58)^b^; New York: 51 (41 to 61)^b^	63	0:01:40	24	32	N/A	3

Primary Outcomes

**Figure 2:**
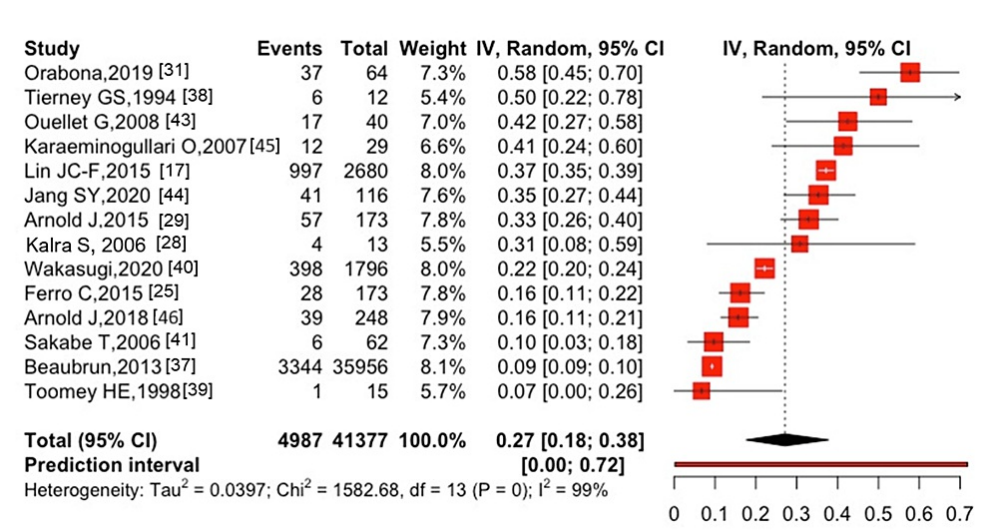
One-year mortality after hip fracture in patients with ESKD ESKD, end-stage kidney disease

Seven studies [14,17,25,26,35,36,46] reported on the one-year mortality rate (range: 215.35-774.0 per 1,000 person-year) after hip and spine fractures, whereas two studies [26,40] revealed the five-year mortality rate (range: 148-194.1 per 1,000 person-year) after hip and spine fractures. In the present study, the mortality rate was estimated with the number of deaths in the numerator and with total person-years in the denominator; however, a meta-analysis was not performed because no more than two studies reported the total person-years in the denominator. The one-year mortality after hip fractures was 27% (95% CI: 18-38%, I^2^ = 98%) in 14 studies [17,25,28,29,31,37-41,43-46], with 4,987 cases of all-cause mortality among 41,377 participants (Figure 2).

**Figure 3:**
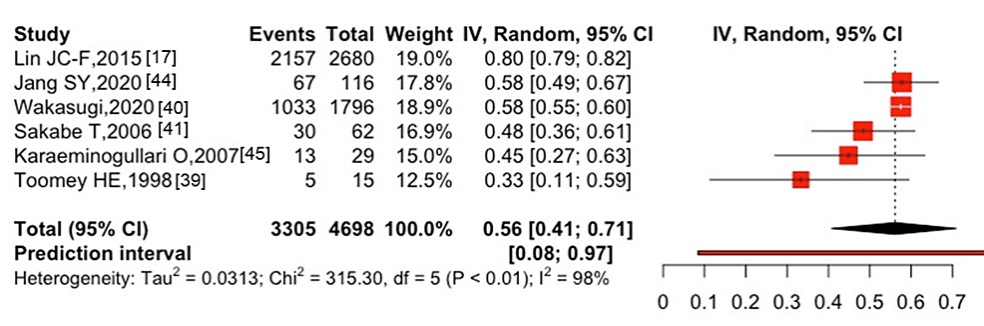
Five-year mortality after hip fracture in patients with ESKD ESKD, end-stage kidney disease

**Figure 4:**
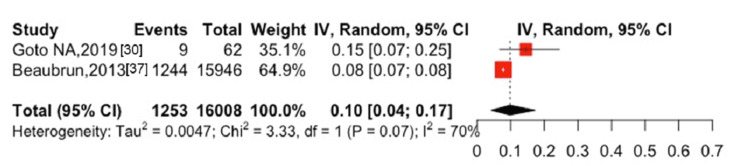
One-year mortality after spinal fracture in patients with ESKD ESKD, end-stage kidney disease

**Figure 5:**
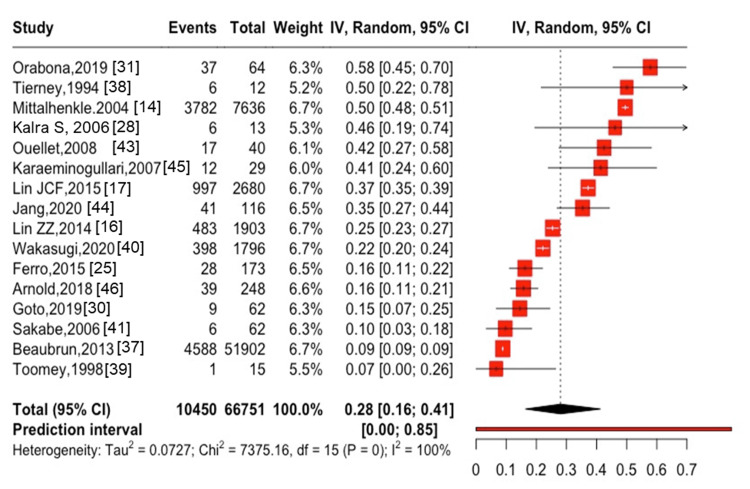
Combined one-year mortality after hip and spinal fractures in patients with ESKD ESKD, end-stage kidney disease

**Figure 6:**
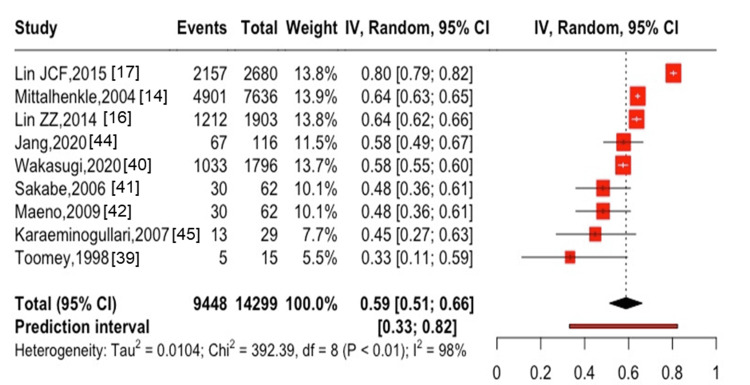
Combined five-year mortality after hip and spinal fractures in patients with ESKD ESKD, end-stage kidney disease

The five-year mortality after hip fractures was 56% (95% CI: 41-71%, I^2^ = 99%) in six studies [17,39-41,44,45], with 3,305 cases of all-cause mortality among 4,698 participants (Figure 3). The one-year mortality after spine fractures was 10% (95% CI: 4-17%, I^2^ = 70%) in two studies [30,37], with 1,253 cases of all-cause mortality among 16,008 participants (Figure 4). Only one study [41] showed the five-year mortality after spine fractures, which reported a five-year mortality of 48.3% (30 cases of death among 62 patients followed). The combined one-year mortality after hip and spine fractures was 28% (95% CI: 16-41%, I^2^ = 100%) in 16 studies [14,16,17,25,28,30,31,37-41,43-46] with 10,450 cases of all-cause mortality among 66,751 participants (Figure 5). The combined five-year mortality after hip and spine fractures was 59% (95% CI: 51-66%, I^2^ = 100%) in nine studies [14,16,17,39-42,44,45], with 9,448 cases of all-cause mortality among 14,299 participants (Figure 6).

Subgroup Analyses

**Figure 7:**
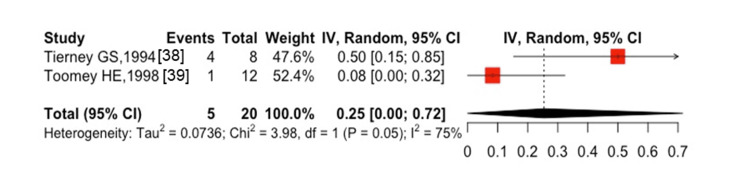
One-year mortality after hip and spinal fractures in female patients with ESKD ESKD, end-stage kidney disease

**Figure 8:**
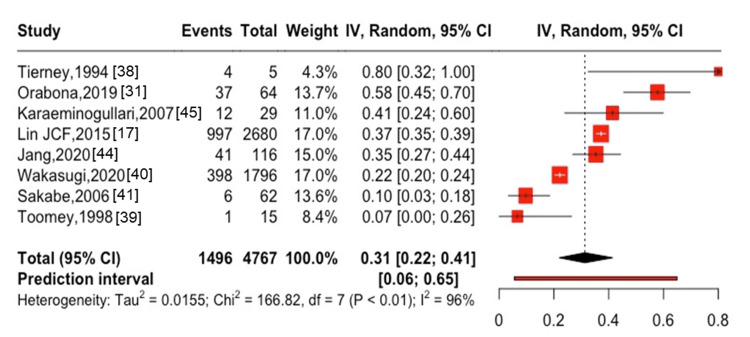
One-year mortality after hip and spinal fractures in patients with hemodialysis

**Figure 9:**
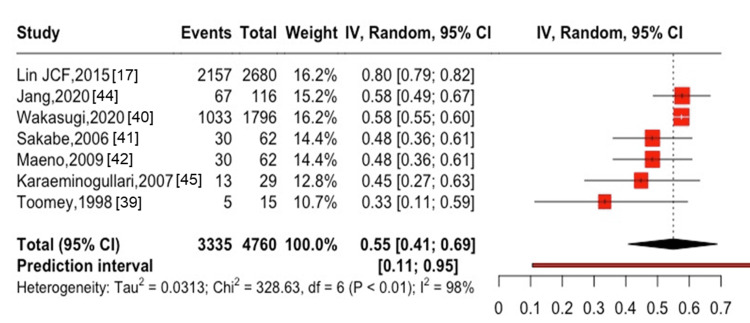
Five-year mortality after hip and spinal fractures in patients with hemodialysis

**Figure 10:**
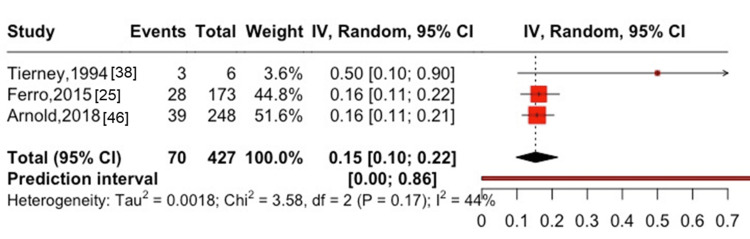
One-year mortality after hip and spinal fractures in patients with post-kidney transplantation

**Figure 11:**
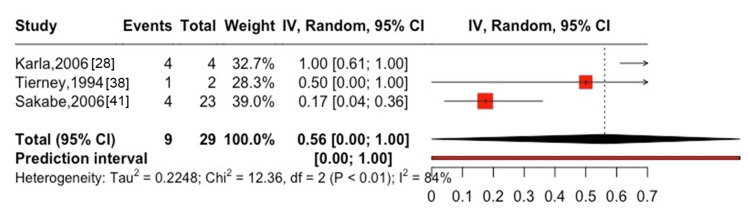
One-year mortality after hip and spinal fractures in patients aged 75 and older

In women, the one-year mortality rate was 20% (95% CI: 0-82%, I^2^ = 54%) in two studies [38,39], with two cases of all-cause mortality among seven participants (Figure 7). In patients on hemodialysis, the one-year mortality rate was 31% (95% CI: 22-41%, I^2^ = 96%) in eight studies [17,31,38-41,44,45], with 1,496 cases of all-cause mortality among 4,767 participants (Figure 8), whereas the five-year mortality rate was 55% (95% CI: 41-69%, I^2^ = 98%) in seven studies [17,39-42,44,45], with 3,335 cases of all-cause mortality among 4,760 participants (Figure 9). Among patients after kidney transplantation, the one-year mortality rate was 15% (95% CI: 10-22%, I^2^ = 44%) in three studies [25,38,46], with 70 cases of all-cause mortality among 427 participants (Figure 10). The one-year mortality in patients aged ≥75 years was 56% (95% CI: 0-100%, I^2^ = 84%) in three studies [28,38,41], with nine cases of all-cause mortality among 29 participants (Figure 11).

Risk of Bias and Certainty of Evidence

The risk-of-bias assessment using the JBI Prevalence Critical Appraisal Tool indicated that 15 studies [13,14,16,17,25-27,30,33,35,36,38,40,42,44], six studies [31,32,34,38,41,46], and five studies [28,29,39,43,45] had a low, moderate, and high risk of bias, respectively (Table 2). A funnel plot asymmetry test was not performed for all outcomes included in the meta-analysis because of the small number of studies included.

**Table 2:** Risk of bias D1: Was the sample frame appropriate to address the target population? D2: Were the study participants sampled appropriately? D3: Was the sample size adequate? D4: Were the study participants and setting described in detail? D5: Was the data analysis conducted with sufficient coverage of the identified sample? D6: Were valid methods used for the identification of the condition? D7: Was the condition measured in a standard and reliable manner for all participants? D8: Was there appropriate statistical analysis? D9: Was the response rate adequate? If not, was a low response rate managed appropriately?

Author	D1	D2	D3	D4	D5	D6	D7	D8	D9	Risk of bias
Tentori [13]	Yes	Yes	Yes	Yes	Yes	Yes	Yes	Yes	Yes	Low
Mittalhenkle [14]	Yes	Yes	Yes	Yes	Yes	Yes	Yes	Yes	Yes	Low
Lin ZZ [16]	Yes	Yes	Yes	Yes	Yes	Yes	Yes	Yes	Yes	Low
Lin JCF [17]	Yes	Yes	Yes	Yes	Yes	Yes	Yes	Yes	Yes	Low
Ferro [25]	Yes	Yes	Yes	Yes	Yes	Yes	Unclear	Yes	Yes	Low
Iseri [26]	Yes	Yes	Yes	Yes	Unclear	Yes	Yes	Yes	Yes	Low
Iseri [27]	Yes	Yes	Yes	Yes	Yes	Yes	Yes	Yes	Yes	Low
Kalra [28]	Not	Yes	Not	Not	Not	Not	Yes	Unclear	Unclear	High
Arnold [29]	Yes	Yes	Yes	Unclear	Unclear	Unclear	Unclear	Unclear	Unclear	High
Goto [30]	Yes	Yes	Yes	Not	Yes	Yes	Yes	Yes	Yes	Low
Orabona [31]	Yes	Yes	Not	Yes	Yes	Yes	Unclear	Yes	Yes	Moderate
Apostolopulos [32]	Not	Unclear	Yes	Not	Yes	Yes	Yes	Unclear	Yes	Moderate
Iseri [33]	Yes	Yes	Yes	Yes	Yes	Yes	Yes	Yes	Yes	Low
Wu [34]	Not	Yes	Yes	Yes	Yes	Not	Yes	Yes	Yes	Moderate
Iseri [35]	Yes	Yes	Yes	Yes	Yes	Yes	Yes	Yes	Yes	Low
Kaneko [36]	Yes	Yes	Yes	Yes	Yes	Yes	Unclear	Yes	Yes	Low
Beaubrun [37]	Yes	Yes	Yes	Yes	Yes	Yes	Yes	Yes	Yes	Low
Tierney [38]	Yes	Yes	Not	Yes	Unclear	Yes	Yes	Unclear	Unclear	Moderate
Toomey [39]	Yes	Yes	Not	Not	Unclear	Yes	Unclear	Unclear	Unclear	High
Wakasugi [40]	Yes	Yes	Yes	Yes	Yes	Yes	Yes	Yes	Yes	Low
Sakabe [41]	Yes	Unclear	Not	Yes	Unclear	Yes	Yes	Yes	Yes	Moderate
Maeno [42]	Yes	Yes	Yes	Yes	Yes	Yes	Yes	Yes	Yes	Low
Ouellet [43]	Yes	Yes	Not	Not applicable	Not applicable	Unclear	Unclear	Unclear	Unclear	High
Jang [44]	Yes	Yes	Yes	Yes	Yes	Yes	Yes	Yes	Yes	Low
Karaeminogullari [45]	Not	Unclear	Not	Yes	Not	Not	Not	Yes	Unclear	High
Arnold [46]	Yes	Yes	Unclear	Yes	Unclear	Yes	Unclear	Yes	Yes	Moderate

Discussion

By performing a systematic review and meta-analysis, we comprehensively and quantitatively analyzed the mortality rates and mortality after hip and spine fractures in patients with ESKD receiving kidney replacement therapy. We found that when patients with ESKD had hip and spine fractures, their mortality rate was high, particularly during the first year after these fractures. Notably, 27% and 56% of patients with ESKD after hip fractures died at one year and five years later, respectively. These results suggest that patients and their family members should be informed of poor prognosis, and clinicians should strictly and appropriately manage the mineral bone disease. Such a high risk of post-fracture mortality among patients with ESKD is consistent with the findings of a previous study by Tentori et al. [13], who utilized data from the Dialysis Outcomes and Practice Patterns Study and reported that the mortality rate was the highest in the first month following the fracture event and that the rate declined thereafter. 

There are several explanations for the higher risk of post-fracture mortality in patients with ESKD than those in the general population. First, patients with ESKD might have been in such a poor condition at the time of fracture occurrence that they were judged intolerable to surgery. Indeed, when patients with ESKD sustain hip fractures, conservative management is chosen in up to 13% of patients [14]. In the general population, however, most patients are treated with surgical repair or arthroplasty [47,48], and nonoperative management is selected for only 2.6% of patients after hip fractures. Furthermore, a previous study [15] using data from the United States Renal Data System showed that patients with fractures who did not undergo surgery at the time of their fracture had a higher all-cause mortality than those who did. This applies to patients without ESKD. A previous study [49] showed that geriatric hip fracture patients who were treated nonoperatively had a higher in-hospital, 30-day, and one-year mortality than a matched group of operatively treated patients. Hence, nonoperative management of hip fractures in patients with ESKD may be related to the decline in mobility and muscle strength, contributing to the high mortality rate [49,50]. Another possible explanation is that patients with ESKD after fractures may experience postoperative complications. The results of several previous studies support this hypothesis by showing that patients undergoing dialysis have an increased risk of infectious and cardiovascular complications after surgery [51,52]. For example, Benjamin et al. [51] reported that patients on dialysis had 2.9- and 1.7-fold higher risks of sepsis and pneumonia, respectively, than non-dialyzed patients within 30 days of hip fracture fixation. Additionally, a previous meta-analysis [52] highlighted that patients on dialysis had a two- to five-fold increase in the odds of postoperative myocardial infarction and stroke, regardless of the surgical procedure. Alternatively, this result may be confounded by indications, and patients who do not undergo surgery are less likely to undergo surgery because of poor health status or comorbidities at the time of fracture occurrence.

We also found that the one-year and five-year mortality rates were 10% and 48%, respectively, when patients with ESKD sustained spine fractures. To our knowledge, this is the first study to systematically evaluate the prognosis after spine fractures in patients with ESKD; however, our findings should be interpreted with caution because the five-year mortality rate solely depends on the results of a study by Maeno et al. [42]. Our results showed that many previous studies on the prognosis of post-spine fractures were limited to short-term observations, and they have reported that hyperkyphosis and vascular calcifications were associated with poor prognosis in patients after spine fracture [30,37,53]. For instance, a prospective multicenter cohort study [30] following incident dialysis for one year reported that thoracic hyperkyphosis and increased curvature of the thoracic spine were associated with a higher risk of all-cause mortality. Moreover, another single-center cohort study [53] showed an association between vertebral fractures (thoracic and lumbar) and higher two-year mortality in female patients receiving hemodialysis. Furthermore, the results of these studies [30,37,53] did not report the cause of death; therefore, it is unclear whether the deaths were due to cardiovascular diseases or other causes. The longitudinal influence of spine fractures on mortality should be further investigated with a focus on the cause of death in future studies.

However, this study had several limitations. First, there may have been some missing studies because we included the outcome term in our search. Because database searches can only identify search terms in titles and abstracts, the inclusion of outcome terms may be subject to outcome reporting bias [54]. Second, we only included studies written in English. Nonetheless, this study included studies reported from Europe, the United States, Canada, and Asian countries, which we believe minimizes selection bias and ensures generalizability. Third, differences in the study design, participant populations, and diagnostic procedure likely contributed to the heterogeneity of our results. Indeed, we could not perform several preplanned subgroup analyses to explain the heterogeneity of our findings, and only a few studies were included. Also, it should be noted that diagnostic procedures for spine fractures vary in the respective articles [30,37,53]. Additionally, several of the included studies did not report important clinical information, such as dialysis vintage, history of cardiovascular diseases, diabetes, or past fractures. This may affect the reliability with which clinicians can apply our estimates. Fourth, several studies involved the same investigators, and it was not possible to completely exclude overlaps in the patient population. Fifth, hip and spine fractures may not be comparable because of differences in treatment and prognosis, but these two conditions are common in patients with ESKD.

## Conclusions

In conclusion, this study showed that patients with ESKD sustaining hip and spine fractures had high post-fracture mortality rates. The one-year mortality was 27% after hip fracture in 14 studies and 10% after spine fracture in two studies. Additionally, the five-year mortality rate was 56% after hip fractures in six studies and 48% after spine fractures in one study. While it remains unclear whether deaths were related to fractures or a consequence of cardiovascular diseases or infections, understanding the prognosis of these types of fractures will contribute to guiding clinical management and fracture prevention in this high-risk population. More importantly, it is essential in advancing care planning and shared decision-making for those receiving kidney replacement therapy, which has recently received a great deal of attention.

## Appendices

Supplementary text: difference between protocol and review

Due to a lack of data, we could not analyze one-year mortality and five-year mortality after fractures for Blacks, patients with cardiovascular diseases, and those with diabetes mellitus.

**Table 3:** PRISMA 2020 checklist PRISMA, Preferred Reporting Items for Systematic Reviews and Meta-Analyses

Section and topic	Item #	Checklist item	Location where item is reported
TITLE			
Title	1	Identify the report as a systematic review.	1
ABSTRACT			
Abstract	2	See the PRISMA 2020 for Abstracts checklist.	1-2
INTRODUCTION			
Rationale	3	Describe the rationale for the review in the context of existing knowledge.	3-4
Objectives	4	Provide an explicit statement of the objective(s) or question(s) the review addresses.	4
METHODS			
Eligibility criteria	5	Specify the inclusion and exclusion criteria for the review and how studies were grouped for the syntheses.	4-5
Information sources	6	Specify all databases, registers, websites, organisations, reference lists, and other sources searched or consulted to identify studies. Specify the date when each source was last searched or consulted.	5
Search strategy	7	Present the full search strategies for all databases, registers, and websites, including any filters and limits used.	Tables S2-S6
Selection process	8	Specify the methods used to decide whether a study met the inclusion criteria of the review, including how many reviewers screened each record and each report retrieved, whether they worked independently, and if applicable, details of automation tools used in the process.	6-7
Data collection process	9	Specify the methods used to collect data from reports, including how many reviewers collected data from each report, whether they worked independently, any processes for obtaining or confirming data from study investigators, and if applicable, details of automation tools used in the process.	7
Data items	10a	List and define all outcomes for which data were sought. Specify whether all results that were compatible with each outcome domain in each study were sought (e.g. for all measures, time points, and analyses), and if not, the methods used to decide which results to collect.	5
	10b	List and define all other variables for which data were sought (e.g. participant and intervention characteristics and funding sources). Describe any assumptions made about any missing or unclear information.	5
Study risk of bias assessment	11	Specify the methods used to assess risk of bias in the included studies, including details of the tool(s) used, how many reviewers assessed each study and whether they worked independently, and if applicable, details of automation tools used in the process.	7-8
Effect measures	12	Specify for each outcome the effect measure(s) (e.g. risk ratio and mean difference) used in the synthesis or presentation of results.	8-9
Synthesis methods	13a	Describe the processes used to decide which studies were eligible for each synthesis (e.g. tabulating the study intervention characteristics and comparing against the planned groups for each synthesis (item #5)).	9
	13b	Describe any methods required to prepare the data for presentation or synthesis, such as handling of missing summary statistics, or data conversions.	9
	13c	Describe any methods used to tabulate or visually display results of individual studies and syntheses.	9
	13d	Describe any methods used to synthesize results and provide a rationale for the choice(s). If meta-analysis was performed, describe the model(s), method(s) to identify the presence and extent of statistical heterogeneity, and software package(s) used.	9
	13e	Describe any methods used to explore possible causes of heterogeneity among study results (e.g. subgroup analysis and meta-regression).	9
	13f	Describe any sensitivity analyses conducted to assess robustness of the synthesized results.	9
Reporting bias assessment	14	Describe any methods used to assess risk of bias due to missing results in a synthesis (arising from reporting biases).	9
Certainty assessment	15	Describe any methods used to assess certainty (or confidence) in the body of evidence for an outcome.	Not applicable
RESULTS			
Study selection	16a	Describe the results of the search and selection process, from the number of records identified in the search to the number of studies included in the review, ideally using a flow diagram.	Figure 1
	16b	Cite studies that might appear to meet the inclusion criteria, but which were excluded, and explain why they were excluded.	10-11
Study characteristics	17	Cite each included study and present its characteristics.	11
Risk of bias in studies	18	Present assessments of risk of bias for each included study.	13
Results of individual studies	19	For all outcomes, present, for each study: (a) summary statistics for each group (where appropriate) and (b) an effect estimate and its precision (e.g. confidence/credible interval), ideally using structured tables or plots.	12
Results of syntheses	20a	For each synthesis, briefly summarise the characteristics and risk of bias among contributing studies.	Table S7
	20b	Present results of all statistical syntheses conducted. If meta-analysis was done, present for each the summary estimate and its precision (e.g. confidence/credible interval) and measures of statistical heterogeneity. If comparing groups, describe the direction of the effect.	Figures 2-6
	20c	Present results of all investigations of possible causes of heterogeneity among study results.	Figures 2-11
	20d	Present results of all sensitivity analyses conducted to assess the robustness of the synthesized results.	Figures 7-11
Reporting biases	21	Present assessments of risk of bias due to missing results (arising from reporting biases) for each synthesis assessed.	43
Certainty of evidence	22	Present assessments of certainty (or confidence) in the body of evidence for each outcome assessed.	Not applicable
DISCUSSION			
Discussion	23a	Provide a general interpretation of the results in the context of other evidence.	13
	23b	Discuss any limitations of the evidence included in the review.	15-16
	23c	Discuss any limitations of the review processes used.	16
	23d	Discuss implications of the results for practice, policy, and future research.	16
OTHER INFORMATION			
Registration and protocol	24a	Provide registration information for the review, including register name and registration number, or state that the review was not registered.	4
	24b	Indicate where the review protocol can be accessed, or state that a protocol was not prepared.	4
	24c	Describe and explain any amendments to information provided at registration or in the protocol.	43
Support	25	Describe sources of financial or non-financial support for the review, and the role of the funders or sponsors in the review.	4
Competing interests	26	Declare any competing interests of review authors.	4
Availability of data, code, and other materials	27	Report which of the following are publicly available and where they can be found: template data collection forms; data extracted from included studies; data used for all analyses; analytic code; any other materials used in the review.	Not applicable

**Table 4:** CENTRAL search strategy CENTRAL, Cochrane Central Register of Controlled Trials ([mh "kidney diseases"] OR [mh "renal replacement therapy"] OR [mh "renal dialysis"] OR [mh "peritoneal dialysis"] OR [mh Hemodiafiltration] OR [mh "hemodialysis, home"] OR (ESRD:ti,ab OR "End Stage Renal Disease":ti,ab OR ESKD:ti,ab OR "End Stage Kidney Disease":ti,ab OR ESKF:ti,ab OR "End Stage Kidney Failure":ti,ab OR ESRF:ti,ab OR "End Stage Renal Failure":ti,ab OR CKD:ti,ab OR "Chronic Kidney Disease":ti,ab OR "Chronic Kidney Failure":ti,ab OR "Renal Transplantation":ti,ab OR CAPD:ti,ab OR CCPD:ti,ab OR APD:ti,ab OR Hemodialysis:ti,ab OR Haemodialysis:ti,ab OR Hemodiafiltration:ti,ab OR Haemodiafiltration:ti,ab)) AND ([mh "femoral fractures"] OR ("Femoral Fracture":ti,ab OR "Femoral Neck Fracture":ti,ab OR "Hip Fracture":ti,ab) OR ([mh "spinal fractures"] OR ("Vertebral Fracture":ti,ab OR "Spine Fracture":ti,ab))) AND ([mh incidence] OR [mh mortality] OR [mh "follow up studies"] OR prognos*:ti,ab,kw OR predict*:ti,ab,kw OR course*:ti,ab,kw) NOT ([mh “Animals”] NOT [mh “Humans”])

#1	[mh “Kidney Diseases”]
#2	[mh “Renal Replacement Therapy”]
#3	[mh “Renal Dialysis”]
#4	[mh “Peritoneal Dialysis”]
#5	[mh “Hemodiafiltration”]
#6	[mh “Hemodialysis, Home”]
#7	#1 OR #2 OR #3 OR #4 OR #5 OR #6
#8	ESRD:ti,ab
#9	“end stage renal disease”:ti,ab
#10	ESKD:ti,ab
#11	“end stage kidney disease”:ti,ab
#12	ESKF:ti,ab
#13	“end stage kidney failure”:ti,ab
#14	ESRF:ti,ab
#15	“end stage renal failure”:ti,ab
#16	CKD:ti,ab
#17	“chronic kidney disease”:ti,ab
#18	“chronic kidney failure”:ti,ab
#19	“renal transplantation”:ti,ab
#20	CAPD:ti,ab
#21	CCPD;ti,ab
#22	APD;ti,ab
#23	“hemodialysis”:ti,ab
#24	“haemodialysis”ti,ab
#25	“hemodiafiltration”:ti,ab
#26	“haemodiafiltration”:ti,ab
#27	#8 OR #9 OR #10 OR #11 OR #12 OR #13 OR #14 OR #15 OR #16 OR #17 OR #18 OR #19 OR #20 OR #21 OR #22 OR #23 OR #24 OR #25 OR #26
#28	#7 OR #27
#29	[mh “Femoral Fractures”]
#30	“femoral fracture”:ti,ab
#31	“femoral neck fracture”:ti,ab
#32	“hip fracture”:ti,ab
#33	#30 OR #31 OR #32
#34	#29 OR #33
#35	[mh “Spinal Fractures”]
#36	“vertebral fracture”:ti,ab
#37	“spine fracture”:ti,ab
#38	#36 OR #37
#39	#35 OR #38
#40	#34 OR #39
#41	"incidence [MeSH: noexp]" OR [mh mortality] OR "follow up studies [MeSH: noexp]" OR prognos*:ti,ab,kw OR predict*:ti,ab,kw OR course*:ti,ab,kw
#42	#28 AND #40 AND #41
#43	[mh “Animals”] NOT [mh “Humans”]
#44	#42 NOT #43

**Table 5:** MEDLINE (via PubMed) search strategy (("kidney diseases"[MeSH Terms] OR "renal replacement therapy"[MeSH Terms] OR "renal dialysis"[MeSH Terms] OR "peritoneal dialysis"[MeSH Terms] OR "Hemodiafiltration"[MeSH Terms] OR "hemodialysis, home"[MeSH Terms] OR ("ESRD"[Title/Abstract] OR "End Stage Renal Disease"[Title/Abstract] OR "ESKD"[Title/Abstract] OR "End Stage Kidney Disease"[Title/Abstract] OR "ESKF"[Title/Abstract] OR "End Stage Kidney Failure"[Title/Abstract] OR "ESRF"[Title/Abstract] OR "End Stage Renal Failure"[Title/Abstract] OR "CKD"[Title/Abstract] OR "Chronic Kidney Disease"[Title/Abstract] OR "Chronic Kidney Failure"[Title/Abstract] OR "Renal Transplantation"[Title/Abstract] OR "CAPD"[Title/Abstract] OR "CCPD"[Title/Abstract] OR "APD"[Title/Abstract] OR "Hemodialysis"[Title/Abstract] OR "Haemodialysis"[Title/Abstract] OR "Hemodiafiltration"[Title/Abstract] OR "Haemodiafiltration"[Title/Abstract])) AND ("femoral fractures"[MeSH Terms] OR ("Femoral Fracture"[Title/Abstract] OR "Femoral Neck Fracture"[Title/Abstract] OR "Hip Fracture"[Title/Abstract]) OR ("spinal fractures"[MeSH Terms] OR ("Vertebral Fracture"[Title/Abstract] OR "Spine Fracture"[Title/Abstract]))) AND ("incidence"[MeSH Terms] OR "mortality"[MeSH Terms] OR "follow up studies"[MeSH Terms] OR "prognos*"[Text Word] OR "predict*"[Text Word] OR "course*"[Text Word])) NOT ("animals"[MeSH Terms] NOT "humans"[MeSH Terms])

#1	Kidney diseases [mh]
#2	Renal Replacement Therapy [mh]
#3	Renal Dialysis [mh]
#4	Peritoneal Dialysis [mh]
#5	Hemodiafiltration [mh]
#6	Hemodialysis, Home [mh]
#7	#1 OR #2 OR #3 OR #4 OR #5 OR #6
#8	ESRD [tiab]
#9	“End Stage Renal Disease” [tiab]
#10	ESKD [tiab]
#11	“End Stage Kidney Disease” [tiab]
#12	ESKF [tiab]
#13	“End Stage Kidney Failure” [tiab]
#14	ESRF [tiab]
#15	“End Stage Renal Failure” [tiab]
#16	CKD [tiab]
#17	“Chronic Kidney Disease” [tiab]
#18	“Chronic Kidney Failure” [tiab]
#19	“Renal Transplantation” [tiab]
#20	CAPD [tiab]
#21	CCPD [tiab]
#22	APD [tiab]
#23	“Hemodialysis” [tiab]
#24	“Haemodialysis” [tiab]
#25	“Hemodiafiltration” [tiab]
#26	“Haemodiafiltration” [tiab]
#27	#8 OR #9 OR #10 OR #11 OR #12 OR #13 OR #14 OR #15 OR #16 OR #17 OR #18 OR #19 OR #20 OR #21 OR #22 OR #23 OR #24 OR #25 OR #26
#28	#7 OR #27
#29	Femoral Fractures [mh]
#30	“Femoral Fracture” [tiab]
#31	“Femoral Neck Fracture” [tiab]
#32	“Hip Fracture” [tiab]
#33	#30 OR #31 OR #32
#34	#29 OR #33
#35	Spinal Fractures [mh]
#36	“Vertebral Fracture” [tiab]
#37	“Spine Fracture” [tiab]
#38	#36 OR #37
#39	#35 OR #38
#40	#34 OR #39
#41	incidence [MeSH: noexp] OR mortality [MeSH Terms] OR follow up studies [MeSH: noexp] OR prognos*[Text Word] OR predict*[Text Word] OR course*[Text Word]
#42	#28 AND #40 AND #41
#43	animals [mh] NOT humans [mh]
#44	#42 NOT #43

**Table 6:** Embase search strategy (ProQuest Dialog)

S1	EMB.EXACT.EXPLODE(“kidney diseases”)
S2	EMB.EXACT.EXPLODE(“renal replacement therapy”)
S3	EMB.EXACT.EXPLODE (“hemodialysis”)
S4	EMB.EXACT.EXPLODE (“peritoneal dialysis”)
S5	EMB.EXACT.EXPLODE (“hemodiafiltration”)
S6	EMB.EXACT.EXPLODE (“home dialysis”)
S7	#1 OR #2 OR #3 OR #4 OR #5 OR #6
S8	ab(ESRD) OR ti(ESRD)
S9	ab(end stage renal disease) OR ti(end stage renal disease)
S10	ab(ESKD) OR ti(ESKD)
S11	ab(end stage kidney disease) OR ti(end stage kidney disease)
S12	ab(ESKF) OR ti(ESKF)
S13	ab(end stage kidney failure) OR ti(end stage kidney failure)
S14	ab(ESRF) OR ti(ESRF)
S15	ab(end stage renal failure) OR ti(end stage renal failure)
S16	ab(CKD) OR ti(CKD)
S17	ab(chronic kidney disease) OR ti(chronic kidney disease)
S18	ab(chronic kidney failure) OR ti(chronic kidney failure)
S19	ab(renal transplantation) OR ti(renal transplantation)
S20	ab(CAPD) OR ti(CAPD)
S21	ab(CCPD) OR ti(CCPD)
S22	ab(APD) OR ti(APD)
S23	ab(hemodialysis) OR ti(hemodialysis)
S24	ab(haemodialysis) OR ti(haemodialysis)
S25	ab(hemofiltration) OR ti(hemofiltration)
S26	ab(haemodiafiltration) OR ti(haemodiafiltration)
S27	S8 OR s9 OR S10 OR S11 OR S12 OR S13 OR S14 OR S15 OR S16 OR S17 OR S18 OR S19 OR S20 OR S21 OR S22 OR S23 OR S24 OR S25 OR S26
S28	S7 OR S27
S29	EMB.EXACT.EXPLODE (“femur fractures”)
S30	ab(femoral fracture) OR ti(femoral fracture)
S31	ab(femoral neck fracture) OR ti(femoral neck fracture)
S32	ab(hip fracture) OR ti(hip fracture)
S33	S30 OR S31 OR S32
S34	S29 OR S33
S35	EMB.EXACT.EXPLODE (“spine fractures”)
S36	ab(vertebral fracture) OR ti(vertebral fracture)
S37	ab(spinal fracture) OR ti(spinal fracture)
S38	S36 OR S37
S39	S35 OR S38
S40	S34 OR S39
S41	EMB.EXACT.EXPLODE (“mortality”)
S42	EMB.EXACT.EXPLODE (“survival”)
S43	EMB.EXACT.EXPLODE (“prognosis”)
S44	S41 OR S42 OR S43
S45	ab(mortality) OR ti(mortality)
S46	ab(survival) OR ti(survival)
S47	ab(prognosis) OR ti(prognosis)
S48	S45 OR S46 OR S47
S49	S44 OR S48
S50	S28 AND S40 AND S49
S51	EMB.EXACT (animal experiment) NOT (EMB.EXACT (human experiment) OR EMB.EXACT (human))
S52	S50 NOT S51

**Table 7:** ICTRP search strategy ICTRP, International Clinical Trials Registry Platform

Conditions: “Kidney Diseases” OR “Renal Replacement Therapy” OR “Renal Dialysis” OR “Peritoneal Dialysis” OR “Hemodiafiltration”
Intervention: “Femoral Fractures” OR “Spinal Fractures”
Recruitment status is ALL.

**Table 8:** ClinicalTrials.gov search strategy

Condition or disease: (Kidney Diseases OR Renal Replacement Therapy OR Renal Dialysis OR Peritoneal Dialysis OR Hemodiafiltration) AND (Femoral Fractures OR Spinal Fractures)
Intervention: Not applicable
Other terms: Mortality OR Death OR Survival OR Prognosis
